# New Triphenylamine-Based Oligomeric Schiff Bases Containing Tetraphenylsilane Moieties in the Backbone

**DOI:** 10.3390/polym11020216

**Published:** 2019-01-27

**Authors:** Patricio A. Sobarzo, Alexis F. González, Eduardo Schott, Luis H. Tagle, Alain Tundidor-Camba, Carmen González-Henríquez, Ignacio A. Jessop, Claudio A. Terraza

**Affiliations:** 1 Research Laboratory for Organic Polymers, Pontificia Universidad Católica de Chile, P.O. Box 306, Post 22, Santiago, Chile; patosobarzzo@aol.com (P.A.S.); afgonzalez1@uc.cl (A.F.G.); ltagle@uc.cl (L.H.T.); atundido@uc.cl (A.T.-C.); 2 Department of Inorganic Chemistry, Pontificia Universidad Católica de Chile, P.O. Box 306, Post 22, Santiago, Chile; edschott@uc.cl; 3 Laboratory of Nanotechnology and Advanced Materials, Universidad Tecnológica Metropolitana, P.O. Box 9845, Post 21, Santiago, Chile; carmenmabel@gmail.com; 4 Laboratorio de Materiales Orgánicos y Poliméricos, Department of Chemistry, Faculty of Chemistry, Universidad de Tarapacá, P.O. Box 7-D, Arica, Chile; Ignacio.jessop@gmail.com; 5 UC Energy Research Center, Pontificia Universidad Católica de Chile, Santiago, Chile

**Keywords:** tetraphenylsilane, triphenylamine, oligomeric Schiff bases, optoelectronic applications

## Abstract

Three new triphenylamine-based oligomeric Schiff bases (polySB1, polySB2 and polySB3) containing tetraphenylsilane core (TPS-core) in the main chain were obtained from TPS-core-based diamines and bis(4-formylphenyl)phenylamine by a high-temperature polycondensation reaction. These new oligomers were structurally characterized by FT-IR, NMR and elemental analysis. All polySBs were highly soluble in common organic solvents, such as chloroform, tetrahydrofuran and chlorobenzene. Samples showed moderate molecular average molecular weight (Mw) and a high thermal stability above 410 °C. Likewise, polySBs showed absorption near 400 nm in the UV-vis range and photoluminescence. The HOMO levels and band-gap values were found in the ranges of −6.06 to −6.18 eV and 2.65–2.72 eV, respectively. The lowest band-gap value was observed for polySB2, which could be attributed to a more effective *π*-conjugation across the main chain. The results suggest that silicon-containing polySBs are promising wide-band-gap semiconductors materials for optoelectronic applications.

## 1. Introduction

In recent years, the design and synthesis of new conjugated polymers have attracted the attention of many research groups due to their excellent optoelectronic properties and due to being versatile semiconductor materials for polymer solar cells (PSCs), polymer light-emitting diodes (PLEDs) and organic field-effect transistors (OFETs) devices [1,2]. Additionally, these materials have been widely studied because they display interesting features such as flexibility, large and fast area processing, light-weight and low fabrication cost compared with traditional materials. The advantages of the conjugated polymers make them an alternative to conventional inorganic-based compounds [3].

Among conjugated polymers, polymeric Schiff bases (polySBs) containing imine bonds (C=N) have been investigated as optoelectronic and electrochemical materials, liquid crystals and metal-catalyst supports among others [4,5,6,7,8,9,10]. Conjugated polySBs are usually synthesized from aromatic diamines and dialdehyde compounds by polycondensation reactions for obtaining aromatic π-systems in the backbone. Furthermore, the sp^2^ hybridization of the nitrogen and carbon atoms of the imine group can extend the conjugation of the polymer chains and increase their hole-transporting ability [11]. Whole aromatic polySBs have interesting properties such as high thermal stability, mechanical strength and semiconducting properties [12,13]. These kinds of polymers are becoming excellent candidates as active materials for PSCs, PLEDs and organic light-emitting diodes (OLEDs), photovoltaic cells and pH sensors, due to their electrochromic properties, among other applications [6,12,14].

Small molecules and polymers bearing triphenylamine (TPA) moieties have been extensively investigated due to their strong electron-donating and hole transporting/injecting ferromagnetic properties [7,13]. TPA-based compounds are used as building blocks for constructing electrochromic materials such as PLEDs, PSCs and electrochromic (ECs) devices due to their absorption/emission, redox activity, charge transport and ferromagnetism properties [15,16,17]. Likewise, the trigonal geometry of the central nitrogen atom in TPA-containing compounds allows obtaining materials with good solubility [15]. In the last decades, research in optoelectronic devices has explored the use of polySBs-containing TPA as donor or acceptor materials in PSCs. Zhang et. al. reported for the first time a TPA-based n-type non-fullerene acceptor copolymers with PTB7-Th as a donor, achieving a power conversion efficiency of 2.2% in a photovoltaic device [18]. PLEDs based on TPA-polymers with carbazole-triphenylamines building-blocks and a cyanophenyl withdrawing group displayed a low turn-on voltage, high luminance and current efficiency, demonstrating that tuning the hole and electron transport properties through suitable donors units could be an effective way to achieve proper materials [19].

Recently, silicon-containing small molecules and polymers have been investigated as materials for optoelectronics applications, gas separation membranes and the synthesis of porous organic frameworks [20,21,22,23,24]. Silicon-containing polymers, particularly tetra-arylsilane (TPS) derivatives, have become attractive moieties for efficient blue OLEDs as a host material due to their high-energy gap and suitable triplet energy level along with their high quantum efficiency in host/blue-phosphorescent OLEDs [25]. Furthermore, polymers with TPS moieties have shown excellent properties for the preparation of materials with wide band-gaps due to the δ-Si structure, which can interrupt the extended π-conjugation achieving high triplet energy materials, high electrochemical, thermal and morphological stability and good film-forming ability [26,27,28]. Furthermore, the non-planar tetrahedral TPS geometry in silicon-containing polymers can effectively suppress the intermolecular interactions enhancing the processability by higher solubility for low-cost processing techniques, such as spin-coating and ink-jet printing desirable for large-area applications [22,27,29,30].

Nowadays, despite the significant importance of solution-processing TPS-containing polymers for advanced applications, there are few reports in the literature [22,25,30]. To the best of our knowledge, there are only two previous reports from silicon-containing polySBs, and both were developed by our research group [29,31]. In this context, the present research was focused on the synthesis, photo–physical and structural characterization of three new conjugated triphenylamine-based polySBs containing TPS moieties in their backbone. The donor effect of TPA-core in the polySBs was studied alternating different tetraphenylsilane moieties (*p*-TPS/*p*-phenyl-TPS/*m*-phenyl-TPS). Thus, this work is the third report of aromatic silicon-containing polySBs and the first report on triphenylamine-based polySBs incorporating silicon atoms by TPS-cores.

## 2. Materials and Methods

### 2.1. Materials

1,4-Dibromobenzene, 4-bromo-*N,N*-bis(trimethylsilyl)aniline, *p*-toluenesulfonic acid monohydrate (PTSA), *n*-butyllithium, anhydrous *N,N*-dimethylacetamide (DMAc), anhydrous calcium sulfate were purchased from Sigma-Aldrich (Milwaukee, WI, USA). 3-Nitrophenylboronic acid, 4-aminophenylboronic acid pinacol ester, bis(4-formylphenyl)phenylamine (TPA) and tetrabutylammonium hexafluorophosphate (TBAHFP) were obtained from AK Scientific, Inc. (San Francisco, USA). Potassium carbonate, chloroform, *n*-hexane, toluene, 1,4-dioxane, dichloromethane and tetrahydrofuran were purchased from Merck (Darmstadt, Germany). Except for THF, which was dried over sodium using benzophenone as indicator under nitrogen atmosphere and distilled immediately before its use, all reagents and solvents were used as received without further purification.

#### 2.1.1. Synthesis and Spectroscopic Characterization of Silylated Precursors and Monomers

Silylated precursors and monomers were obtained by following previous reports. Thus, bis(4-bromophenyl)diphenylsilane (P1) and bis(4-aminophenyl)diphenylsilane (A1) were prepared by a bromo-lithium exchange reaction of the respective bromine compound and then treated with dichlorodiphenylsilane [22,29,31,32]. On the other hand, bis(3’-nitro-[1,1’-biphenyl]-4-yl)diphenylsilane (P2) and bis(4’-amino-[1,1’-biphenyl]-4-yl)diphenylsilane (A2) were synthesized by Suzuki-Miyaura cross-coupling reactions from P1, the respective boronic acid or boronate ester derivative and palladium catalyst [31,33,34]. A3 monomer (bis(3’-amino-[1,1’-biphenyl]-4-yl)diphenylsilane) was obtained from reduction of P2 using hydrazine/Pd-carbon system. 

Bis(4-bromophenyl)diphenylsilane (P1)

Yield: 59%. M.P. (°C): 165–166. ^1^H NMR (CDCl_3_, 400 MHz) δ ppm: 7.51 (m, 8H), 7.42 (m, 2H), 7.38 (m, 8H). ^13^C NMR (101 MHz, CDCl_3_) δ ppm: 137.85, 136.23, 133.01, 132.64, 131.22, 130.04, 128.14, 125.00. ^29^Si NMR (79 MHz, CDCl_3_) δ ppm: −14.08. FT-IR (KBr pellets) cm^−1^: 3065 ν(H–C, arom.), 1566–1472 ν(C=C, arom.), 1526, 1107 ν(Si–C arom.), 1064 ν(C–Br), 810 γ(*p*-di-subst.), 726 γ(*mono*-subst.).

Bis(3’-nitro-[1,1’-biphenyl]-4-yl)diphenylsilane (P2)

Yield: 75%. M.P. (°C): 177–179. ^1^H NMR (400 MHz, CDCl_3_) δ ppm: 8.50 (s, 2H), 8.23 (d, J = 8.1 Hz, 2H), 7.95 (d, J = 7.7 Hz, 2H), 7.75 (d, J = 7.7 Hz, 4H), 7.65 (dd, J = 18.4 Hz; 10.7 Hz, 10H), 7.46 (dt, J = 14.1 Hz; 7.0 Hz, 6H). ^13^C NMR (101 MHz, CDCl_3_) δ ppm: 148.94, 142.70, 140.00, 137.32, 136.50, 134.71, 133.58, 133.17, 130.12, 129.95, 128.26, 126.81, 122.46, 122.18. ^29^Si NMR (79 MHz, CDCl_3_) δ ppm: −14.35. FT-IR (KBr pellets) cm^−1^: 3065, 3016 ν(H–C, arom.), 1529, 1340 ν(N–O), 1420 ν(C=C arom.), 1530, 1126 ν(Si–C arom.), 827 γ(*p*-di-subst.), 803 γ(*m*-di-subst.), 702 γ(*mono*-subst.).

Bis(4-aminophenyl)diphenylsilane (A1)

Yield: 40%. M.P. (°C): 205-207. ^1^H NMR (400 MHz, CDCl_3_) δ ppm: 7.58 (d, J = 6.9 Hz, 4H), 7.38 (m, 10H), 6.69 (d, J = 8.1 Hz, 4H), 3.75 (s, 4H). ^13^C NMR (101 MHz, CDCl_3_) δ ppm: 147.71, 137.85, 136.46, 135.89, 129.28, 127.79, 122.63, 114.69. ^29^Si NMR (79 MHz, CDCl_3_) δ ppm: −14.90. FT-IR (KBr pellets) cm^−1^: 3455, 3361 ν(N–H), 3017 ν(C–H, arom.), 1619, 1596, 1504 ν(C=C, arom.), 1525, 1113 ν(Si–C), 820 γ(*p*-di-subst.), 704 γ(*mono*-subst.).

Bis(4’-amino-[1,1’-biphenyl]-4-yl)diphenylsilane (A2)

Yield: 73%. M.P. (°C): 106–107. ^1^H NMR (400 MHz, CDCl_3_) δ ppm: 7.66 (d, J = 3.2 Hz, 8H), 7.59 (d, J = 7.6 Hz, 4H), 7.44 (dt, J = 13.7 Hz; 7.2 Hz, 10H), 6.76 (d, J = 8.0 Hz, 4H), 3.72 (s, 4H). ^13^C NMR (101 MHz, CDCl_3_) δ ppm: 146.22, 142.24, 136.97, 136.56, 134.69, 131.79, 131.21, 129.67, 128.14, 128.00, 125.86, 115.51. ^29^Si NMR (79 MHz, CDCl_3_) δ ppm: −14.51. FT-IR (KBr pellets) cm^−1^: 3448, 3378 ν(N–H), 3016 ν(H–C, arom.), 1618, 1494, 1425 ν(C=C, arom.), 1524, 1186 ν(Si–C arom.), 813 γ(*p*-di-subst.), 738 γ(*mono*-subst.).

Bis(3’-amino-[1,1’-biphenyl]-4-yl)diphenylsilane (A3)

Yield: 91%. M.P. (°C): 98-99. ^1^H NMR (400 MHz, CDCl_3_) δ ppm: 7.49 (t, J = 6.2 Hz, 8H), 7.42 (d, J = 7.5 Hz, 4H), 7.25 (dt, J = 13.7 Hz; 6.7 Hz, 6H), 7.04 (t, J = 7.7 Hz, 2H), 6.85 (d, J = 7.6 Hz, 2H), 6.75 (s, 2H), 6.48 (d, J = 7.8 Hz, 2H), 3.47 (s, 4H). ^13^C NMR (101 MHz, CDCl_3_) δ ppm: 146.79, 142.54, 142.17, 136.88, 136.53, 134.37, 132.99, 129.83, 129.76, 128.04, 126.66, 117.78, 114.49, 113.97. ^29^Si NMR (79 MHz, CDCl_3_) δ ppm: −14.40. FT-IR (KBr pellets) cm^−1^: 3443, 3373 ν(N–H), 3020 ν(H–C, arom.), 1593, 1480, 1425 ν(C=C, arom.), 1490, 1111 ν(Si–C arom.), 825 γ(*m*-di-subst.), 738 γ(*mono*-subst.).

#### 2.1.2. Synthesis and Structural Characterization of PolySBs

All oligomer synthesis was carried out during 24 hours under a nitrogen atmosphere and at high temperature using PTSA as catalyst and anh. calcium sulfate as a water trap. Briefly, in a three-necked round-bottom flask with nitrogen gas inlet, TPA (0.301 g, 1 mmol), PTSA (60 mg, 0.32 mmol) and anh. calcium sulfate (80 mg, 0.59 mmol) were dissolved in anh. DMAc (5 mL) and warm following by slowly adding the aromatic silylated-diamine solution A1, A2 or A3 (1 mmol in 5 mL of anh. DMAc). The reaction mixture was heated at 60 °C for 1.5 h, and then raised at 120 °C for 20 hours. The oligomer solution was then cooled down to room temperature and poured into a methanol-water mixture (1:1 *v*/*v*, 100 mL). The mixture was stirred for 2 hours and the precipitate was collected by filtration and washed with water. The resulting solid was washed thoroughly with hot methanol in a Soxhlet apparatus for 48 hours and were then dried under vacuum at 70 °C for 24 hours.

PolySB1

Yield: 40%, yellow powder. ^1^H NMR (400 MHz, CDCl_3_) δ ppm: 9.86 (CHO terminal), 8.42 (CH=N), 7.89-6.57 (C–H, arom.), 5.54 (N–H terminal). ^13^C NMR (101 MHz, CDCl_3_) δ ppm: 191 (CHO terminal), 159 (C=N), 154 (C–N=C), 141 (C–N, TPA moiety), 135–114 (C, arom.). ^29^Si NMR (79 MHz, CDCl_3_) δ ppm: −14.46. FT-IR (KBr on ATR) cm^-1^: 3026 ν(H–C, arom.), 2881, 2836 ν(=C–H, terminal) 1690 ν(C=O, terminal), 1585 ν(C=N), 1500, 1427 ν(C=C, arom.), 1318 ν(C–N), 1528, 1163 ν(Si–C arom.), 827 γ(*p*-di-subst.), 699 γ(*mono*-subst.). Elem. Anal. Calcd. for (C_44_H_33_N_3_Si)n (631.56)n: C, 83.67%, H, 5.23%, N, 6.65%. Found: C, 78.60 %, H, 5.90%, N, 7.60%.

PolySB2

Yield: 92%, yellow powder. ^1^H NMR (400 MHz, CDCl_3_) δ ppm: 9.87 (CHO terminal), 8.42 (CH=N), 7.95–6.57 (H arom.). ^13^C NMR (101 MHz, CDCl_3_) δ ppm: 191 (CHO terminal), 159 (C=N), 153 (C–N=C), 141 (C–N, TPA moiety), 138–113 (C, arom.). ^29^Si NMR (79 MHz, CDCl_3_) δ ppm: −14.46. FT-IR (KBr in ATR) cm^−1^: 3030 ν(H–C, arom.), 2982, 2946 ν(=C–H, terminal.) 1690 ν(C=O, terminal), 1587 ν(C=N), 1504 ν(C=C, arom.), 1318 v(C–N), 1526, 1111 ν(Si–C arom.), 814 γ(*p*-di-subst.), 700 γ(*mono*-subst.). Elem. Anal. Calcd. for (C_56_H_41_N_3_Si)n (783.68)n: 85.82%, H, 5.23%, N, 5.36%. Found: C, 83.10%, H, 6.30%, N, 5.50%.

PolySB3

Yield: 83%, yellow powder. ^1^H NMR (400 MHz, CDCl_3_) δ ppm: 9.87 (CHO terminal), 8.47 (CH=N), 7.93-6.63 (H, arom.). ^13^C NMR (101 MHz, CDCl_3_) δ ppm: 191 (CHO terminal), 159 (C=N), 153 (C–N=C), 141 (C–N, TPA moiety), 137–113 (C, arom.). ^29^Si NMR (79 MHz, CDCl_3_) δ ppm: −14.45. FT-IR (KBr on ATR) cm^−1^: 3060 ν(H–C, arom.), 1690 ν(C=O, terminal), 1588 ν(C=N), 1523, 1425 ν(C=C, arom.), 1313 ν(C–N), 1523, 1107 ν(Si–C arom.), 827 γ(*p*-di-subst.), 704 γ(*mono*-subst.). Elem. Anal. Calcd. for (C_56_H_41_N_3_Si)n (783.68)n: 85.79%, H, 5.27%, N, 5.36%. Found: C, 82.80%, H, 6.41%, N, 5.74%.

Measurements

^1^H, ^13^C and ^29^Si NMR spectra were recorded on a Bruker Advance III-400 MHz spectrometer (Bruker Corporation, Karlsruhe, Germany) in CDCl_3_. Infrared spectra (FT-IR) were measured on a JASCO FT-IR 4200 spectrometer (Jasco, Easton, MD, USA) using KBr pellets or an ATR Jasco ATR PRO 450-S device (Jasco, Easton, MD, USA). Elemental analyses were acquired on a Thermo Finnigan Flash EA 1112 equipment (Jung Instrument Gmbh, Viersen, Germany). UV-Vis spectra were acquired on a PerkinElmer Lambda 35UV/VIS spectrometer (PerkinElmer, Waltham, MA, USA) and photoluminescent spectra were recorded on a Jasco FP-6200 Spectrofluorometer (Jasco, Easton, MD, USA) using a solution of oligomers in CHCl_3_ (25 mg L^−1^). Thermal analyses were conducted on a Mettler Toledo TGA/SDTA 851 apparatus at a heating rate of 10 °C min^−1^ in nitrogen atmosphere. Differential scanning calorimetry (DSC) were carried out in a Mettler Toledo DSC821 (Mettler Toledo, Greifensee, Switzerland) at heating and cooling rates of 10 °C min^−1^ and 30 °C min^−1^, respectively. Gel permeation chromatography (GPC) experiments were performed on a Wyatt Technology Dawn EOS HPLC (Wyatt Technology, Santa Barbara, CA, USA) with an Optilab DSP differential refractometer index detector, using THF as eluent at flow rate of 1.0 mL min^−1^ at 25 °C and calibrated with a series of monodisperse polystyrene standards. Samples contained 2.5 mg L^−1^ in THF and were filtered through a 0.45 µm nylon filter. Cyclic voltammetry (CV) was carried out on CH Instrument 760E electrochemical analyzer using a three-electrode cell (platinum disc was used as a working electrode, Ag/Ag^+^ as a reference electrode and a platinum wire as counter electrode) and calibrated against the ferrocene/ferrocenium (Fc/Fc^+^) redox couple. All measurements were performed in 0.1 M solution of tetrabutylammonium-hexafluorophosphate (Bu_4_N^+^PF^6−^) in dichloromethane as a supporting electrolyte with a scan rate of 100 mV s^−1^ (Further information can be found in the Supplementary Materials).

## 3. Results and Discussion

### 3.1. Synthesis and Characterization of Diamines

**Scheme 1:**
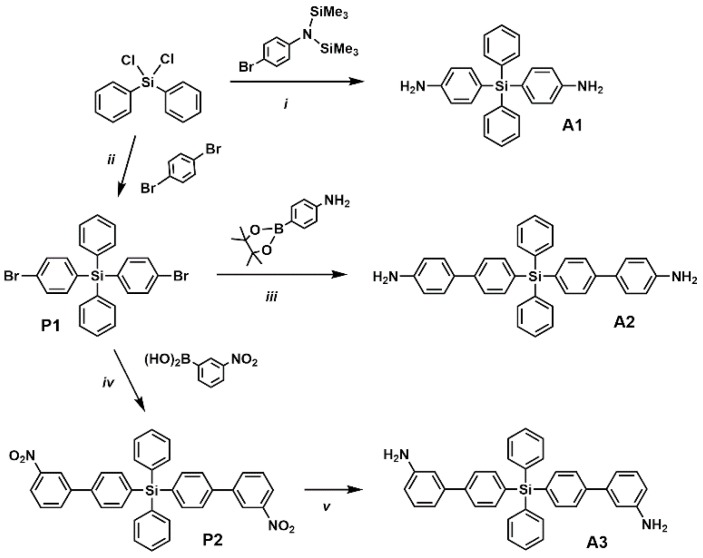
Synthetic route of precursors and monomers; i: 1) *n*-BuLi, anh. THF, 2) HCl (g), 3) NaOH aq. ii: *n*-BuLi, anh. Et_2_O. iii: Pd(PPh_3_)_4_, K_2_CO_3_, toluene/EtOH/H_2_O. iv: PdCl_2_(PPh_3_)_2_, K_2_CO_3_, 1,4-dioxane/H_2_O v: Pd/C, N_2_H_4_.

Scheme 1 shows the synthetic routes for the diamine monomers A1-3. A1 was prepared by a lithium-bromine exchange reaction using 4-bromo-*N,N*-bis(trimethylsilyl)aniline and diphenyldichlorosilane modifying a previously reported methodology [29,33,34]. This last reagent was also used to prepare P1 by a lithium–bromine exchange reaction with 1,4-dibromobenzene. On the other hand, the diamine A2 and the precursor P2 were synthetized by a Suzuki–Miyaura cross-coupling reaction using P1 and 4-aminophenylboronic acid pinacol ester or 3-nitrophenylboronic acid as starting materials, respectively, according to previous reports [29,31,35,36]. Finally, the reduction of P2 using N_2_H_4_ as a reducing agent and Pd/C as a catalyst allows obtaining the diamine A3.

**Figure 1:**
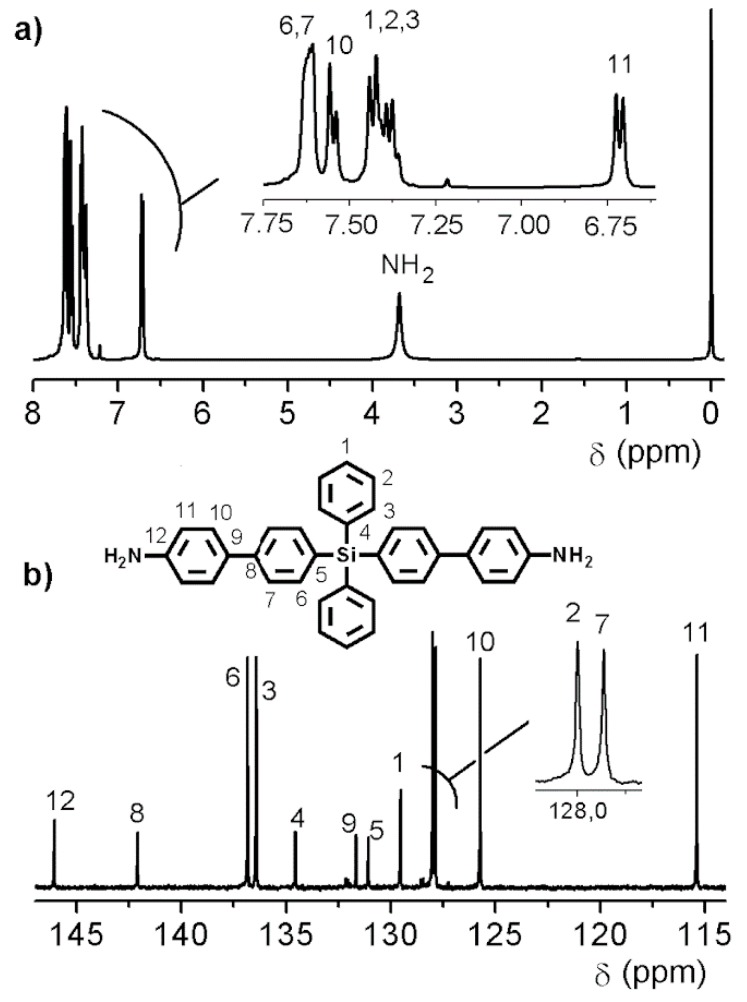
NMR spectra (CDCl_3_) of diamine A2. (**a**) ^1^H NMR, (**b**) ^13^C NMR.

Diamine monomers showed common bands in the FT-IR spectra (Figures S2 and S3); N–H stretching bands at 3450 and 3370 cm^−1^ and also sharp bands at 1490–1530 and 1110–1130 cm^−1^, which were attributed to the stretching and scissoring Si–C arom. vibrations; respectively [37]. The NMR spectra carried out from CDCl_3_ solutions (Figure 1, Figures S1 and S4) showed all expected ^1^H, ^13^C, and ^29^Si signals. For example, the A2 ^1^H NMR spectra (Figure 1) showed a signal centered among 3.5–3.7 ppm for the amino hydrogens, while in the ^13^C NMR analysis, the signal at the lowest magnetic field was assigned to the carbons bearing the amine groups (C8 in A1, C12 in A2 and C13 in A3). The presence of the silicon atom bonded to four phenyl rings was confirmed by a signal at around −14 ppm in the ^29^Si NMR spectra [38].

### 3.2. Synthesis and Structural Characterization of Oligomers

**Scheme 2:**
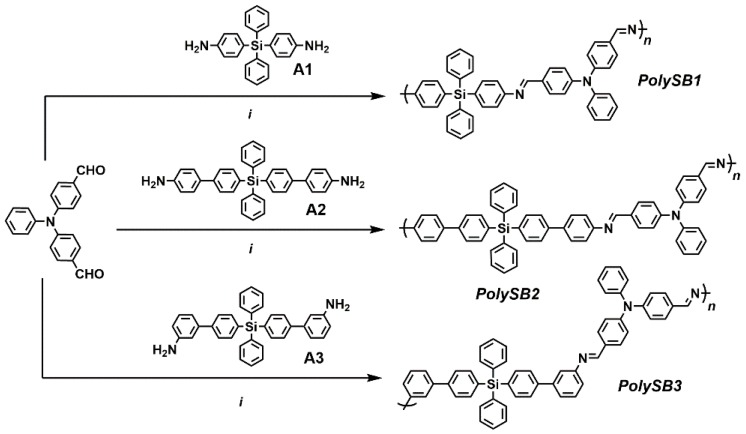
Synthetic routes of oligomeric Schiff bases; i: anh. DMAc, PTSA, 24 h, 120 °C.

PolySBs were prepared from a commercial aromatic dialdehyde (bis(4-formylphenyl)phenylamine) and three silylated diamines containing TPS-cores (A1-A3). All samples were obtained by a high-temperature polycondensation reaction using *p*-toluenesulfonic acid (PTSA) as a catalyst in anhydrous DMAc (Scheme 2). The polymerizations were carried out under N_2_ atmospheric conditions [29,39].

**Figure 2:**
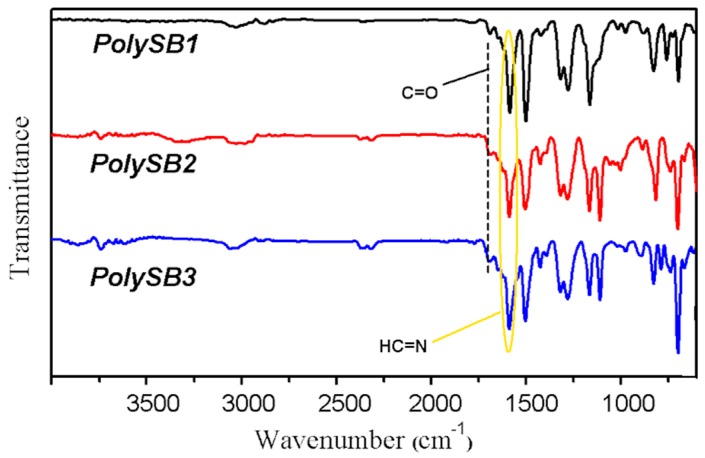
ATR/FT-IR spectra of polySBs.

The proposed structure for the polySBs were confirmed by FT-IR, NMR spectroscopy and elemental analysis. The FT-IR spectra (Figure 2) exhibited an absorption band at about 1590 cm^−1^, corresponding to the imine group. Bands associated with N–H stretching vibrations of diamines were not observed, which means that the polymerization reactions were complete. Absorption bands at about 3040 and 1500 cm^−1^ were attributed to H–C arom. and C=C stretching vibrations of aromatic rings, respectively, while the signals at 1490–1520 cm^−1^ and 1160 cm^−1^ were assigned to Si–C arom. stretching and scissoring vibrations, respectively. Also, a low-intensity absorption band was observed at around 1690 cm^−1^, probably due to the carbonyl (C=O) stretching vibrations of end aldehyde groups of the oligomeric chains [29].

**Figure 3:**
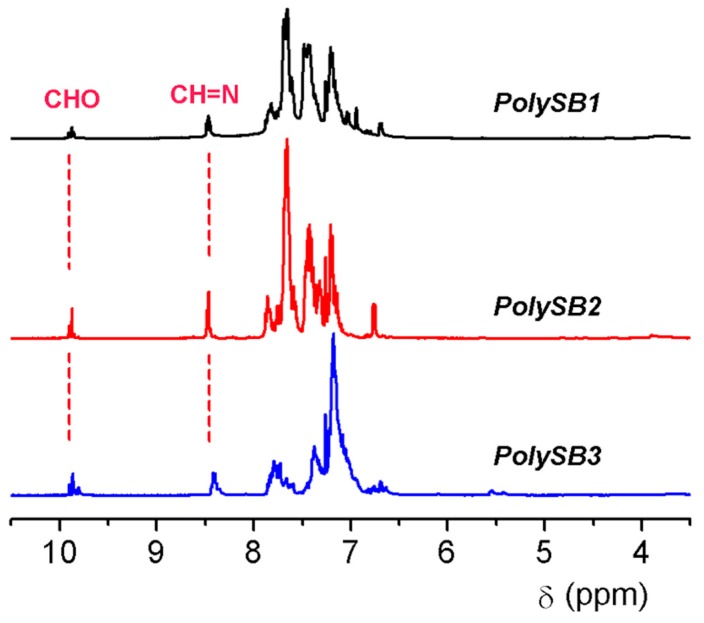
Aromatic region of the ^1^H NMR spectra (CDCl_3_, 400 MHz) of polySBs.

NMR spectra of polySBs also confirmed the proposed structure for the repeat units. In the ^1^H NMR spectra (Figure 3), the imine hydrogen of the monomer linker moieties appears at about 8.5 ppm. The aromatic hydrogens were observed like complex signals in the range of 7.95–6.57 ppm. In accordance with FT-IR observations, all spectra show a signal around 9.9 ppm which is attributable to the terminal aldehyde units.

The imine linkages were also observed in the ^13^C NMR spectra (Figure S5) at about 160 ppm and 140 ppm. Intricate pattern of signals among 139–110 ppm was assigned to the carbon atoms of the C=C bonds of the multiple aromatic systems present in the chains, while the terminal aldehyde moieties were evidenced at 191 ppm. On the other hand, ^29^Si NMR spectra of all polySBs show a distinct peak at around −14.5 ppm, which is due to the silicon atom of the TPS-core moiety [29,31,40]. Finally, the elemental analysis of the oligomers showed results that are in agreement with the structure of the proposed repeat units.

### 3.3. Solubility

The incorporation of both TPS-core and propeller-like structure of TPA units favored polySBs solubility in common organic solvents. The results of the solubility test are summarized in Table S1. As is known, the incorporation of TPA moieties in small molecules or polymers can improve their solubility [7,41,42]. Moreover, the natural twisted tetrahedron geometry of the TPS-core could effectively prevent the intermolecular interactions between the polymeric chains, increasing their solubility [41,42]. Thus, the polySBs were also soluble in low-boiling-point solvents, such as chloroform and THF, making them suitable solution-processable materials which is highly desirable for optoelectronic applications.

### 3.4. Thermal Properties and Molecular Weight

**Figure 4:**
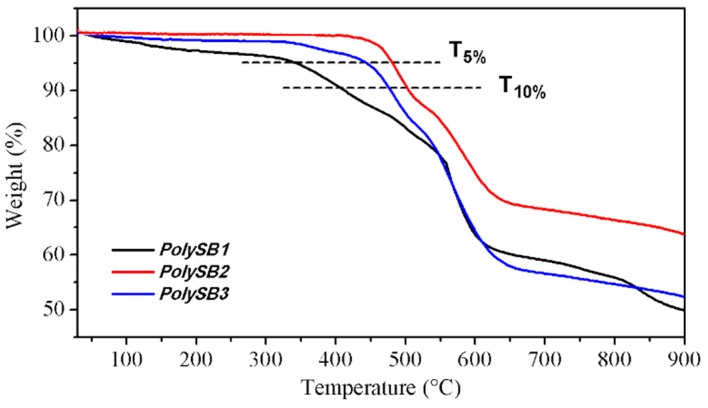
Thermal curves of polySBs with a heating rate of 10 °C min^−1^ in a nitrogen atmosphere.

The thermal properties of oligomers were investigated through thermal and differential scanning calorimetry (DSC) analyses (Figure 4, Figure S6 and Table 1). Figure 4 shows the analysis curve of polySBs performed at a heating rate of 10 °C min^−1^ in a nitrogen atmosphere. All samples showed thermal stability with T_10%_ values over 400 °C and their DTGA results indicated complex processes of decomposition with two marked steps (Figure S6a). The first decomposition temperature could be related to the TPA portion degradation (C–N bonds) and the second one would correspond to the total decomposition of the aromatic rings. It is clear that the incorporation of imine and TPS as a rigid and bulky unit enhanced the thermal stability without losing solubility of the new materials.

The thermal properties of polySBs strongly depend on their chemical structure particularly on the high aromatic rings content in the backbone. All polySBs showed an initial decomposition at 5% weight loss between 340–480 °C. The temperature at 10% weight loss considered for the assumption of thermal stability was observed within the range of 410–500 °C (Table 1). PolySB2 and polySB3, derived from the isomeric biphenylic silylated-diamines A2 and A3; respectively, showed higher T_10%_ values regarding polySB1, based on the phenylic silylated-diamine A1. On the other hand, polySB2 evidenced a T_10%_ 27 °C higher than polySB3, probably due to the *m*-di-substituted structure of A3 which affected the packing of oligomer chains. The char yield values were similar among the samples, and they are in accordance with results already obtained for the analysis of silylated-poly(azomethine)s developed under a nitrogen atmosphere [29,31].

As seen in Table 1 and Figure S6b, the *T*_g_ values were also dependent on the aromatic content on the chains and the effect of the di-substitution on the phenyl rings. Thus, the highest *T*_g_ value of the series corresponds to polySB2 that contains biphenilic moieties and *p*-substitutions in their diamine-monomer. The higher internal rigidity of the polySB2 than polySB1 and polySB3, and their high packing forces explains these results [29].

The molecular weight of the polySBs were measured through gel permeation chromatography analysis (GPC), with mono-disperse polystyrene samples as standard and THF as eluent (Table S2). The weight-average molecular weight (Mw) were 3.9 × 103, 4.5 × 103 and 1.4 × 104 g moL^−1^ for polySB1, polySB2 and polySB3; respectively, while the (Mn) values allowed us to establish the polymerization degree for the samples: dimer for polySB1 and trimer for polySB2 and polySB3. We have obtained similar results in previous work by preparing several silylated-poly(azomethine)s [29,31]. On the other hand, the polydispersity index (PDI) was in the range of 2.17–5.57, with a clear trend to increase with the increment of the molecular weight values. The higher molecular weight of polySB3 would be related to the incorporation of *m*-phenyl di-substituted units which would increase the flexibility of the small-chain and, therefore, their mobility in solution during the polycondensation reaction which would favors the incorporation of the new repeat units and as result an increase in the molecular weight can be achieved [43].

### 3.5. Optical and Electrochemical Properties

**Figure 5:**
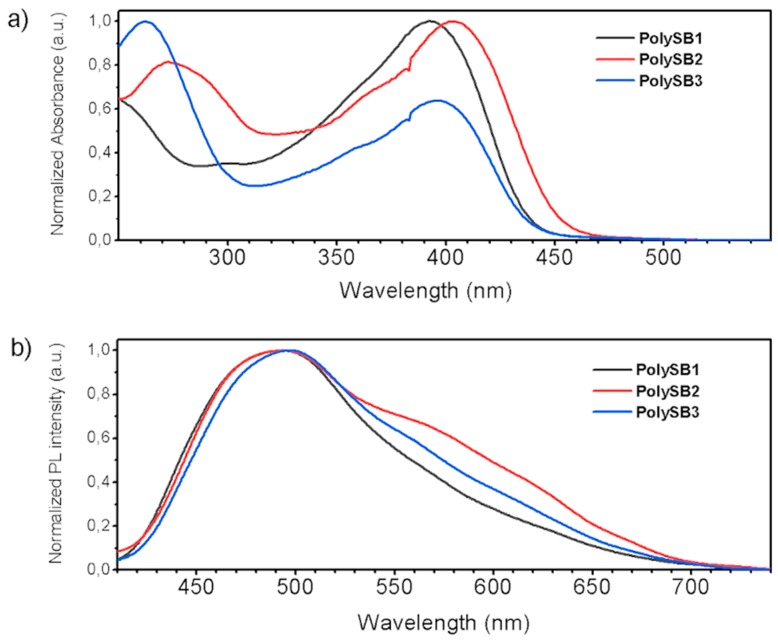
(**a**) Absorption (UV-vis) and (**b**) photoluminescence (PL) spectrum of polySBs in CH_2_Cl_2_.

The optical properties of polySBs were studied through UV-vis absorption and photoluminescence measurements using diluted polymer solutions. As shown in Figure 5a, all polySBs exhibited two well-defined UV-vis absorption bands at around 270 and 400 nm corresponding to π–π* and n–π* transitions of the conjugated system and both TPA and imine moieties, respectively [7,44,45]. PolySB2 exhibited the highest maxima absorption at 473 nm (λ onset, Table 2). The results suggest that the absorption of the polySBs are closely related to the content of aromatic rings and the substitution pattern (polySB1 < polySB3 < polySB2). Thus, polySB2, with a *p*-substitution in their biphenyl moiety, shows a red-shifted maximum absorption band (403 nm) which would be attributed to the difference of dihedral angle between the phenyl and biphenyl moieties. Furthermore, a higher degree of effective conjugation lengths can enhance the degree the π-electronic delocalization in the biphenyl-imine-triphenylamine segments [7,44,46].

According to the values summarized in Table 2, the absorption values of the polySBs are closely related to the structure of the monomers (polySB1 < polySB3 < polySB2). PolySB2, with a *p*-substitution in its biphenyl moiety, exhibited the most red-shifted absorption band (at 403 nm). On the other hand, polySB3 presented slightly similar absorption maximum to polySB1, which could be attributed to the decrease of the π-orbitals overlapping between the polySB3 chains, due to the less linear structure of the main chain regarding polySB2 together with the difference of dihedral angle between the phenyl and biphenylic moieties of polySB3 in comparison with polySB1. This decrease of the orbital overlapping could also explain the lower intensity of the band at about 400 nm in comparison with the aromatic band at about 270 nm of polySB3. Therefore, a greater degree of effective conjugation lengths enhanced the degree the π-electronic delocalization in the biphenyl-imine-triphenylamine segments [7,44,46]. Table 2 also shows the calculated molar absorption coefficient (ε) for the samples, by indicating the ability of each polySB to capture light at the wavelength of analysis.

The optical band-gaps were estimated from de absorption spectra edges according to Kaya et al. [47] and the values were in the range of 2.62–2.75 eV (Table 2). As expected, polySB2 showed the lower band-gap (2.62 eV) and polySB3 showed a slightly lower band-gap than polySB1 (~0.04 eV). 

The lower band-gap of polySB2 is related to the increase of aromatic content in the diamine units regarding to polySB1, owing to phenyl ring with *p*-di-substitution pattern bond to TPS-core (2.75 to 2.62 eV). Likewise, the incorporation of *m*-di-substitution design of monomer in polySB3 induced a lower band-gap value as well as polySB2 (2.75 to 2.71 eV). On the other hand, polySB1 shows the higher band-gap, which would be associated to the lower conjugation lengths in the main chain mainly due to TPS-core linked to TPA unit through imine group [44,48].

The photoluminescence spectra of the polySBs were recorded at room temperature from diluted solutions using dichloromethane as a solvent (Figure 5b) and we have used the UV-vis absorption maximum as exciting-light wavelength. As seen in Figure 5b, all samples showed a broad PL peaks in the range of 410–650 nm, by following the same shift absorption behavior. When the absorption and emission spectra of the samples are compared, the more rigid backbone of polySB2 promotes the smallest Stokes shift [49]. According to PL maximum values (Table 2), the polySBs emit cyan light and, therefore, could be explored as OLEDs active materials in future researches.

**Figure 6:**
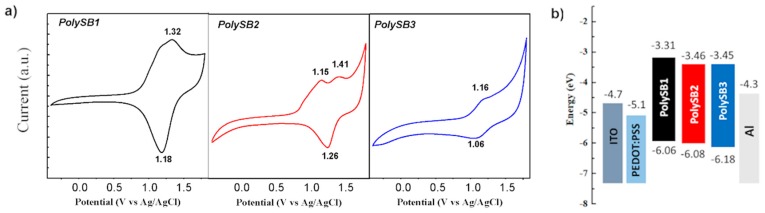
Cyclic voltammograms of polySBs in 0.1 M Bu_4_NPF_6_ in dichloromethane, at a scan rate of 100 mV s^−1^ (**a**) and energy levels (**b**).

The electrochemical properties of polySBs were investigated by cyclic voltammetry (CV). All measurements were performed on a polycrystalline platinum disk as a working electrode in dichloromethane polymer solutions. The voltammograms of the polySBs (Figure 6a and Figure S7) showed oxidative and reductive peaks. For PolySB1 and polySB3, single anodic peaks were observed at 1.32 V and 1.16 V; respectively. Distinctively, polySB2 shows two well-defined oxidative peaks (1.41 V and 1.15 V). The oxidative peaks are attributed to the redox process of the nitrogen atom from TPA-cores, which could generate a radical cation that is stabilized by resonance (Figure S8). The second oxidative peak observed for polySB2 would be the result of a structural arrangement from the radical recombination [7,50,51].

The single anodic peak for polySB1 and polySB3 would indicates that charge carriers formation was hampered because their less-conjugated and less-linear structures, respectively, which is consistent with the high potential required to achieve the doping process or the radical cation formation [52]. All polySBs showed an increase of current and slightly cathodic shifted of the oxidation-peaks when more cycles were applied (Figure S7), proving semiconductor properties of the oligomers [53]. On the other hand, the reductive peaks at 1.18, 1.26, and 1.06 for polySB1, polySB2, and polySB3, respectively, would reflect the de-doping or radical recombination process.

The HOMO levels of polySBs were calculated using the following equation: HOMO (eV) = −[Eox – E1/2 (ferrocene) + 4.8], where Eox is the onset oxidation potential of the polymers, and E1/2(ferrocene) is the onset oxidation potential of ferrocene vs Ag/Ag^+^ as a reference electrode [53]. Thus, the HOMO energy values found for the tested samples were between −6.06 and 6.18 eV (Table 2, Figure 6b) which were lower than HOMO levels of others silicon-containing polymers or those triphenylamine based-polymers [18,19,28,54]. These low-lying HOMO levels could be related to the presence of the TPS-cores in the main chain which displays no significant difference concerning the moiety’s structure (*p*-TPS/*p*-phenyl-TPS/*m*-phenyl-TPS) bonding to TPA-cores by imine linkage. Besides, these low-lying HOMO energy levels give them a series of interesting properties such as high photodegradation stability, large hole mobility and potentially high open circuit voltages to the devices based on them [55]. The lowest HOMO level was observed for polySB3, while polySB1 and polySB2 show a slight difference (0.02 eV). It is clear that the incorporation of δ-Si atoms plays a key role to achieve wide band-gap materials through interruption of π-conjugation along the polymer chains, while the effect of different groups attached directly to the TPS-cores can be an effective way of tuning the HOMO and LUMO energy levels. Furthermore, the attracting electron behavior of the imine linkage and the TPS-core moieties could increase the oxidation potential of the polySBs in a similar way to those observed for small TPA-compounds and other polymeric systems [50,54].

The LUMO levels were calculated from the difference between the HOMO energy levels and the corresponding band-gaps and were estimated in the range of −3.31 to −3.46 eV. These results suggest that the new materials could be used together with proper acceptor materials, like fullerene or non-fullerene polymers, as an active layer for PSCs device. Thus, these LUMO energies could provide a suitable LUMO levels offset between polySBs and PCBMs (−4.0 eV) for an effective exciton dissociation at the interface between donor and acceptor materials [52,56].

## 4. Conclusions

Three new conjugated silicon-containing polySBs bearing triphenylamine moieties were synthesized from different silylated aromatic diamines and bis(4-formylphenyl)phenylamine by high-temperature polycondensation reactions. All polySBs showed solubility in common organic solvents, even in chloroform and THF, and high thermal resistance with *T*_10%_ values above 410 °C and *T*_g_ values ranging from 250 °C to 270 °C. The photophysical measurements of polySBs showed absorption near 400 nm and emission behavior with similar band-gaps in the range of 2.62–2.75 eV. All polySBs showed HOMO energy levels at about −6.1 eV, which result in low values in relation to other reported triphenylamine-based polymers. In this sense, polySB3, containing biphenyl moieties and *m*-di-substitutions, displayed the lowest HOMO level at −6.18 eV. Therefore, the results obtained for these new silicon-containing polySBs with TPA units in the backbone provide a starting point to develop a new class of materials that could be potentially used in optoelectronic applications, and motivates future researches regarding the modification of the chemical structures.

## Figures and Tables

**Table 1:** Thermal analysis of polySBs.

Polymer	*T*_5%_ [°C] ^a^	*T*_10%_ [°C] ^a^	Char Yield [%] ^b^	*T*_g_ [°C] ^c^
polySB1	341	411	50	266
polySB2	478	502	64	334
polySB3	442	475	52	257

^a^*T*_5%_ and T_10%_: temperature at 5% and 10% weight loss, respectively. ^b^ Residual weight at 900 °C in nitrogen atmosphere. ^c^ *T*_g_: Glass transition temperature.

**Table 2:** Optical and electrochemical properties of polySBs.

PolySBs	λ_max_^abs^ [nm]	λ_onset_^abs^ [nm]	λ_max_^em^ [nm]	*E*_g_^opt^ [eV] ^a^	*E*_HOMO_ [eV] ^b^	*E*_LUMO_ [eV] ^c^	(M^−1^ cm^−1^) ^d^
polySB1	394	451	493	2.75	−6.06	−3.31	18,257
polySB2	403	473	498	2.62	−6.08	−3.46	15,047
polySB3	397	457	499	2.71	−6.18	−3.45	13,667

^a^ Estimated from *E*g^opt^ = 1242/λ_onset_. ^b^ Estimated from *E*_onsetox_. ^c^ Calculated from ELUMO = (EHOMO + *E*g^opt^). ^d^ Molar absorption coefficient.

## Supplementary Materials

The following are available online at http://www.mdpi.com/2073-4360/11/2/216/s1, Figure S1: NMR spectra (CDCl_3_) of diamine **A1**. a) ^1^H NMR and b) ^13^C NMR, Figure S2: FT-IR spectrum (KBr pellets) of diamine A2, Figure S3: FT-IR spectrum (KBr pellets) of diamine A3, Figure S4: ^1^H NMR spectra (CDCl_3_, 400 MHz) of diamine A3, Figure S5: ^13^C NMR spectra (CDCl_3_, 101 MHz) of polySBs, Figure S6: Thermal analysis of polySBs. a) DTGA and b) DSC, Figure S7: Cyclic voltammograms of polySBs at 15 cycles measurements in 0.1 M Bu_4_NPF_6_ in dichloromethane, at a scan rate of 100 mV s^−1^, Figure S8: Redox process of the nitrogen atom from TPA-cores; Table S1: Solubility of polySBs, Table S2: GPC results for the polySBs.

## References

[1] Zhang J., Tan H.-S., Guo X., Facchetti A., Yan H. (2018). Material insights and challenges for non-fullerene organic solar cells based on small molecular acceptors. Nat. Energy.

[2] Ye L., Hu H., Ghasemi M., Wang T., Collins B.A., Kim J.-H., Jiang K., Carpenter J.H., Li H., Li Z. (2018). Quantitative relations between interaction parameter, miscibility and function in organic solar cells. Nat. Mater..

[3] Kang H., Kim G., Kim J., Kwon S., Kim H., Lee K. (2016). Bulk-Heterojunction Organic Solar Cells: Five Core Technologies for Their Commercialization. Adv. Mater..

[4] Grigoras M., Catanescu C.O. (2004). Imine Oligomers and Polymers. J. Macromol. Sci. Part C Polym. Rev..

[5] Iwan A., Schab-Balcerzak E., Korona K.P.M., Grankowska S., Kaminska M. (2013). Investigation of optical and electrical properties of new aromatic polyazomethine with thiophene and cardo moieties toward application in organic solar cells. Synth. Met..

[6] Kamacı M., Avcı A., Kaya I. (2015). The crosslinked poly(azomethine-urethane)s containing *o*-hydroxyazomethine: Tunable multicolor emission, photophysical and thermal properties. Prog. Org. Coat..

[7] Ma X., Wu Y., Wen H., Niu H., Wang C., Qin C., Bai X., Lei L., Wang W. (2016). Immobilized polyazomethines containing triphenylamine groups on ITO: Synthesis and acidochromic, electrochemical, electrochromic and photoelectronic propertie. RSC Adv..

[8] Iwan A. (2015). An overview of LC polyazomethines with aliphatic–aromatic moieties: Thermal, optical, electrical and photovoltaic properties. Renew. Sustain. Energy Rev..

[9] Yea H., Jiang F., Li H., Xu Z., Yin J., Zhu H. (2017). Facile synthesis of conjugated polymeric Schiff base as negative electrodes for lithium ion batteries. Electrochim. Acta.

[10] Verde-Sesto E., Maya E.M., Lozano A.E., de la Campa J.G., Sanchez F., Iglesias M. (2012). Novel efficient catalysts based on imine-linked mesoporous polymers for hydrogenation and cyclopropanation reactions. J. Mater. Chem..

[11] Petrus M.L., Bouwer R.K.M., Lafont U., Athanasopoulos S., Greenham N.C., Dingemans T.J. (2014). Small-Molecule Azomethines: Organic Photovoltaics via Schiff Base Condensation Chemistry. J. Mater. Chem. A.

[12] Iwan A., Sek D. (2008). Processible polyazomethines and polyketanils:from aerospace to light-emitting diodes and other advanced applications. Prog. Polym. Sci..

[13] Hu Y.C., Chen J.C., Yen H.J., Lin K.Y., Yeh J.M., Chen W.C., Liou G.S. (2012). Novel triphenylamine-containing ambipolar polyimides with pendant anthraquinone moiety for polymeric memory device, electrochromic and gas separation applications. J. Mater. Chem..

[14] Nowak E.M., Sanetra J., Grucela M., Schab-Balcerzak E. (2015). Azomethine naphthalene diimides as component of active layers in bulk heterojunction solar cells. Mater. Lett..

[15] Yen H.J., Liou G.S. (2012). Solution-processable triarylamine-based electroactive high performance polymers for anodically electrochromic applications. Polym. Chem..

[16] Hacioglu S.O., Toksabay S., Sendur M., Toppare L. (2014). Synthesis and electrochromic properties of triphenylamine containing copolymers: Effect of π-bridge on electrochemical properties. J. Polym. Sci. A Polym. Chem..

[17] Xiong J., Wei Z., Xu T., Zhang Y., Xiong C., Dong L. (2017). Polytriphenylamine derivative with enhanced electrochemical performance as the organic cathode material for rechargeable batteries. Polymer.

[18] Wang X., Lv L., Gu W., Wang X.-I., Dong T., Yang Z., Cao H., Huang H. (2017). Novel triphenylamine-based copolymers for all-polymer solar cells. Dyes Pigm..

[19] Lin Y., Chen Z.-K., Ye T.-L., Dai Y.-F., Ma D.-G., Ma Z., Liu Q.-D., Chen Y. (2010). Novel fluorene-based light-emitting copolymers containing cyanophenyl pendants and carbazole-triphenylamines: Synthesis, characterization and their PLED application. Polymer.

[20] Chen H.-Y., Hou J., Hayden A.E., Yang H., Houk K.N., Yang Y. (2009). Silicon Atom Substitution Enhances Interchain Packing in a Thiophene-Based Polymer System. Adv. Mater..

[21] Liu Y., Lai J.Y.L., Chen S., Li Y., Jiang K., Zhao J., Li Z., Hu H., Ma T., Lin H. (2015). Efficient non-fullerene polymer solar cells enabled by tetrahedron-shaped core based 3D-structure small-molecular electron acceptors. J. Mater. Chem. A.

[22] Moncada J., Terraza C.A., Tagle L.H., Coll D., Ortiz P., Pérez G., de la Campa J.G., Álvarez C., Tundidor-Camba A. (2017). Synthesis, characterization and studies of properties of six polyimides derived from two new aromatic diamines containing a central silicon atom. Eur. Polym. J..

[23] Li L., Ren H., Yuan Y., Yu G., Zhu G. (2014). Construction and adsorption properties of porous aromatic frameworks via AlCl3-triggered coupling polymerization. J. Mater. Chem. A.

[24] Kim G.W., Yang D.R., Kim Y.C., Yang H.I., Fan J.G., Lee C.-H., Chai K.I., Kwon J.H. (2017). Di(biphenyl)silane and carbazole based bipolar host materials for highly efficient blue phosphorescent OLEDs. Dyes Pigm..

[25] Liu H., Cheng G., Hu D., Shen F., Lv Y., Sun G., Yang B., Lu P., Ma Y. (2012). A Highly Efficient, Blue-Phosphorescent Device Based on a Wide-Bandgap Host/FIrpic: Rational Design of the Carbazole and Phosphine Oxide Moieties on Tetraphenylsilane. Adv. Funct. Mater..

[26] Yeh H.-C., Chien C.-H., Shih P.-I., Yuan M.-C., Shu C.-F. (2008). Polymers Derived from 3,6-Fluorene and Tetraphenylsilane Derivatives: Solution-Processable Host Materials for Green Phosphorescent OLEDs. Macromolecules.

[27] Sun D., Zhou X., Li H., Sun X., Zheng Y., Ren Z., Ma D., Bryce M.R., Yan S. (2014). A versatile hybrid polyphenylsilane host for highly efficient solution-processed blue and deep blue electrophosphorescence. J. Mater. Chem. C.

[28] Li X., Bai Q., Li J., Lu F., Sun X., Lu P. (2018). Synthesis and properties of wide bandgap polymers based on tetraphenylsilane and their applications as hosts in electrophosphorescent devices. New J. Chem..

[29] Tundidor-Camba A., González-Henríquez C.M., Sarabia-Vallejos M.A., Tagle L.H., Hauyón R.A., Sobarzo P.A., González A., Ortiz P.A., Maya E.M. (2018). Silylated oligomeric poly(ether-azomethine)s from monomers containing biphenyl moieties: Synthesis and characterization. RSC Adv..

[30] Cai M., Xiao T., Hellerich E., Chen Y., Shinar R., Shinar J. (2011). High-Effi ciency Solution-Processed Small Molecule Electrophosphorescent Organic Light-Emitting Diodes. Adv. Mater..

[31] Tundidor-Camba A., González-Henríquez C.M., Sarabia-Vallejos M.A., Tagle L.H., Sobarzo P.A., González A., Hauyón R.A., Mariman A.P., Terraza C.A. (2018). Diphenylsilane-containing linear and rigid whole aromatic poly(azomethine)s. Structural and physical characterization. Polymer.

[32] Fei T., Cheng G., Hu D.H., Lu P., Ma Y.G. (2009). A wide band gap polymer derived from 3,6-carbazole and tetraphenylsilane as host for green and blue phosphorescent complexes. J. Polym. Sci. Part A Polym. Chem..

[33] Jung S.O., Kim Y.-H., Kwon S.K., Oh H.-Y., Yang J.-H. (2007). New hole blocking material for green-emitting phosphorescent organic electroluminescent devices. Org. Electron..

[34] Pratt J.R., Massey W.D., Pinkerton F.H., Thames S.F. (1975). Organosilicon compounds. XX. Synthesis of aromatic diamines via trimethylsilyl-protecting aniline intermediates. J. Org. Chem..

[35] Ueno A., Kitawaki T., Chida N. (2008). Total Synthesis of (±)-Murrayazoline. Org. Lett..

[36] Thompson A.E., Hughes G., Batsanov A.S., Bryce M.R., Parry P.R., Tarbit B. (2005). Palladium-Catalyzed Cross-Coupling Reactions of Pyridylboronic Acids with Heteroaryl Halides Bearing a Primary Amine Group:  Synthesis of Highly Substituted Bipyridines and Pyrazinopyridines. J. Org. Chem..

[37] González-Henríquez C.M., Terraza C.A., Sarabia M. (2014). Theoretical and Experimental Vibrational Spectroscopic Investigation of Two R1R2-Diphenylsilyl-Containing Monomers and Their Optically Active Derivative Polymer. J. Phys. Chem. A.

[38] Terraza C.A., Tagle L.H., Concha F., Poblete L. (2007). Synthesis and characterization of new bi-functional monomers based on germarylene or silarylene units: 4,4′-(R1R2-silylene)bis(phenyl chloroformates) and 4,4′-(R1R2-germylene)bis(phenyl chloroformates). Des. Monomers Polym..

[39] Iwan A., Boharewicz B., Parafiniuk K., Tazbir I., Gorecki L., Sikora A., Filapek M., Schab-Balcerzak E. (2014). New Air-Stable Aromatic Polyazomethines with Triphenylamine or Phenylenevinylene Moieties towards Photovoltaic Application. Synth. Met..

[40] Terraza C.A., Tagle L.H., Tundidor-Camba A., González-Henríquez C.M., Sarabia-Vallejos M.A., Coll D. (2016). Synthesis and characterization of aromatic poly(ether-imide)s based on bis(4-(3,4-dicarboxyphenoxy)phenyl)-R,R-silane anhydrides (R = Me, Ph)–spontaneous formation of surface micropores from THF solutions. RSC Adv..

[41] Wen H., Niu H., Li B., Ma X., Bai X., Zhang Y., Wang W. (2015). Synthesis and Acidochromic, Electrochromic Properties of Schiff Bases Containing Furan and Triphenylamine Units. Synth. Met..

[42] Liou G., Lin H., Hsieh Y., Yang Y. (2007). Synthesis and Characterization of Wholly Aromatic Poly(azomethine)s Containing Donor–Acceptor Triphenylamine Moieties. Polym. Sci. Part A Polym. Chem..

[43] Liu H., Bai Q., Yao L., Hu D., Tang X., Shen F., Zhang H., Gao Y., Lu P., Yang B. (2014). Solution-Processable Hosts Constructed by Carbazole/PO Substituted Tetraphenylsilanes for Efficient Blue Electrophosphorescent Devices. Adv. Funct. Mater..

[44] Sanchez C.O., Schott E., Zarate X., MacLeod-Carey D., Sobarzo P., Gatica N. (2015). Effect of triphenylamine as electron-donor evenly spaced in 2, 4, 6 and 8 thiophene units of the main chain: Synthesis and properties. Polym. Bull..

[45] Iwan A., Palewicz M., Chuchmała A., Gorecki L., Sikora A., Mazurek B., Pasciak G. (2012). Opto(Electrical) Properties of New Aromatic Polyazomethines with Fluorene Moieties in the Main Chain for Polymeric Photovoltaic Devices. Synth. Met..

[46] Marin L., Bejan A., Ailincai D., Belei D. (2017). Poly(azomethine-phenothiazine)s with efficient emission in solid state. Eur. Polym. J..

[47] Yıldırım M., Kaya I. (2012). Electrochemical syntheses and characterizations of poly(2-aminobenzothiazole)s. Synth. Met..

[48] Zhao Q., Zhang W., Fan Z., Li J., Chen X., Luo G., Zhang X. (2015). Synthesis and characterization of high triplet energy polyfluorene bearing m-tetraphenylsilane segment as a polymer host for green phosphorescent polymer light emitting diodes. Synth. Met..

[49] Lee J., Rajeeva B.B., Yuan T., Guo Z.-H., Lin Y.-H., Al-Hashimi M., Zheng Y., Fang L. (2016). Thermodynamic synthesis of solution processable ladder polymers. Chem. Sci..

[50] Ma X., Niu H., Wen H., Wang S., Lian Y., Jiang X., Wang C., Bai X., Wang W. (2015). Synthesis, electrochromic, halochromic and electro-optical properties of polyazomethines with a carbazole core and triarylamine units serving as functional groups. J. Mater. Chem. C.

[51] Sánchez C.O., Bèrnede J.C., Cattin L., Makha M., Gatica N. (2014). Schiff base polymer based on triphenylamine moieties in the main chain. Characterization and studies in solar cells. Thin Solid Films.

[52] Jessop I.A., Zamora P.P., Díaz F.R., del Valle M.A., Leiva A., Cattin L., Makha M., Bernede J.C. (2012). New Polymers Based on 2,6-di(thiophen-2-yl)aniline and 2,2′-(thiophen-2,5-diyl)dianiline Monomers. Preparation, Characterization and Thermal, Optical, Electronic and Photovoltaic Properties. Int. J. Electrochem. Sci..

[53] Jo J.W., Jung J.W., Wang H.-W., Kim P., Russell T.P., Jo W.H. (2014). Fluorination of Polythiophene Derivatives for High Performance Organic Photovoltaics. Chem. Mater..

[54] Zhong Z., Wang X., Guo T., Cui J., Ying L., Peng J., Cao Y. (2018). Crosslinkable triphenylamine-based hole-transporting polymers for solution-processed polymer light-emitting diodes. Org. Electron..

[55] Yang X., Li Y., Wang X. (2012). Semiconducting Polymer Composites: Principles, Morphologies, Properties and Applications.

[56] Brabec C.J., Gowrisanker S., Halls J.J.M., Laird D., Jia S.J., Williams S.P. (2010). Polymer-fullerene bulk-heterojunction solar cells. Adv. Mater..

