# Supplementary material for: New Triphenylamine-Based Oligomeric Schiff Bases Containing Tetraphenylsilane Moieties in the Backbone
Source: Polymers (Basel). 2019 Jan 27;11(2):216. doi: 10.3390/polym11020216 (PMC6419043; doi:10.3390/polym11020216)
Supplement: Supplementary file 1 [file polymers-11-00216-s001.pdf]

# Supplementary Materials: New Triphenylamine-Based Oligomeric Schiff Bases Containing Tetraphenylsilane Moieties in the Backbone

**Patricio A. Sobarzo, Alexis F. González, Eduardo Schott, Luis H. Tagle, Alain Tundidor-Camba, Carmen González-Henríquez, Ignacio A. Jessop and Claudio A. Terraza**

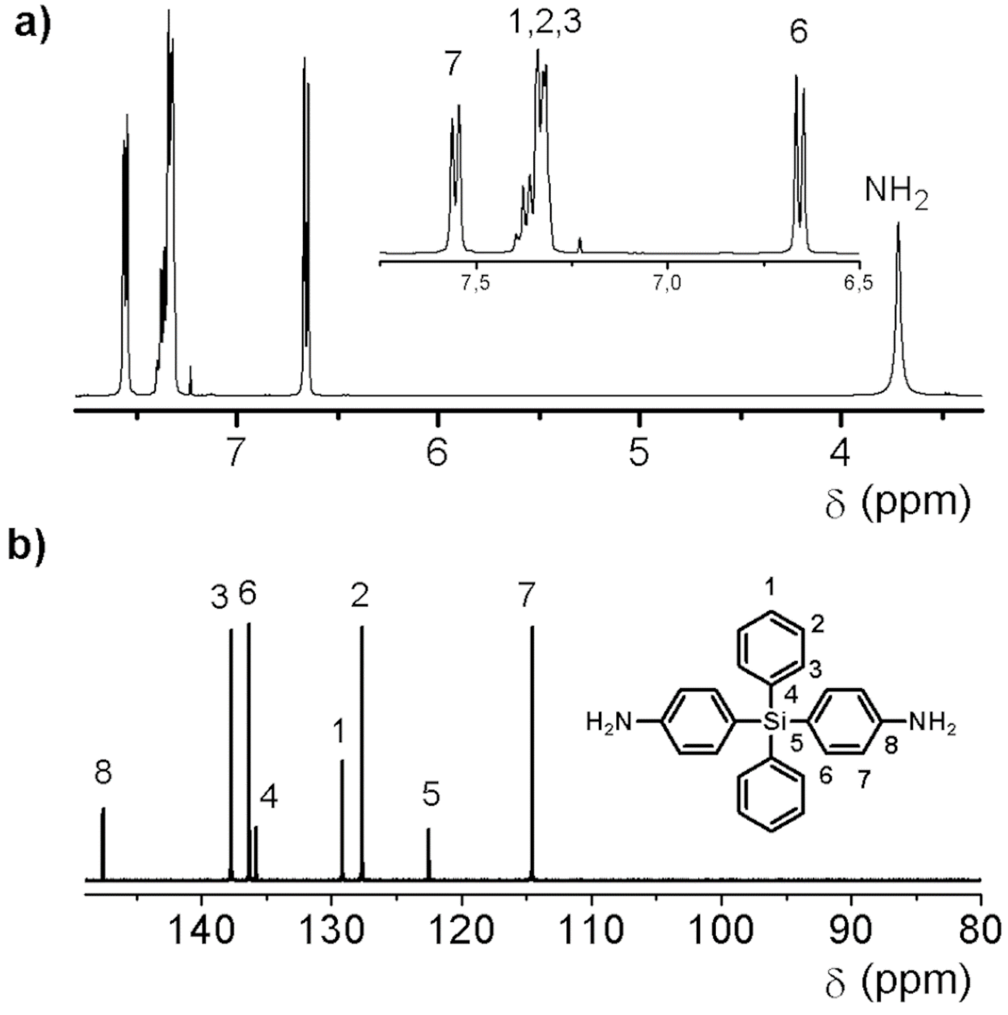


**Figure S1.** NMR spectra ($CDCl_3$) of diamine **A1**. **a**) $^1H$ NMR and **b**) $^{13}C$ NMR.

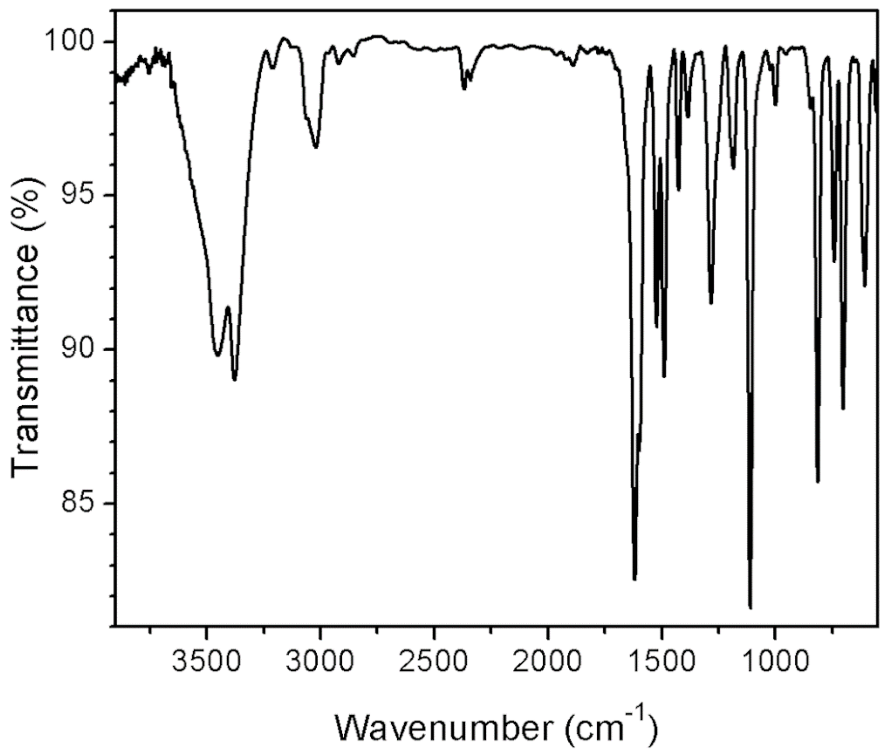


**Figure S2.** FT-IR spectrum (KBr pellets) of diamine **A2**.

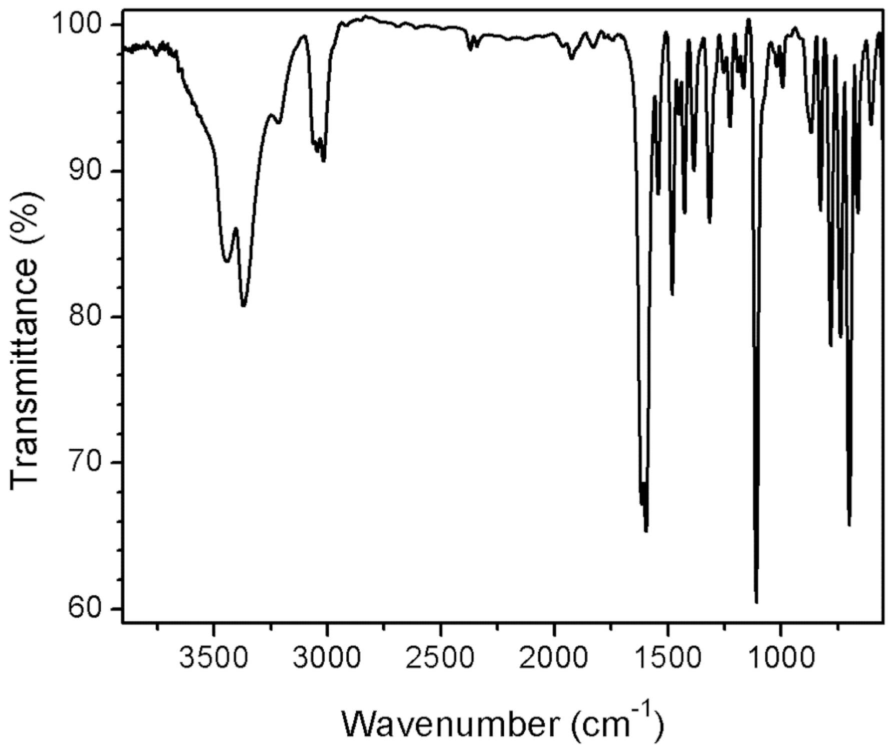


**Figure S3.** FT-IR spectrum (KBr pellets) of diamine **A3**.

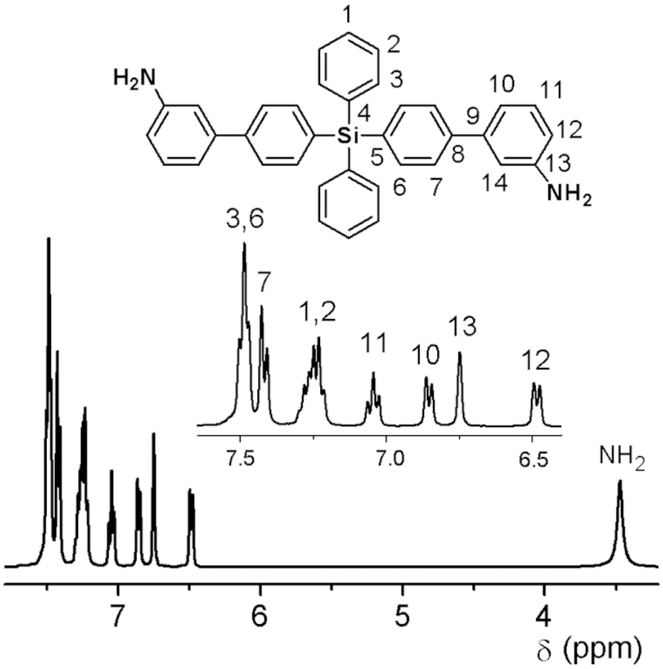


**Figure S4.** $^{1}H$ NMR spectra ($CDCl_3$, 400 MHz) of diamine **A3**.

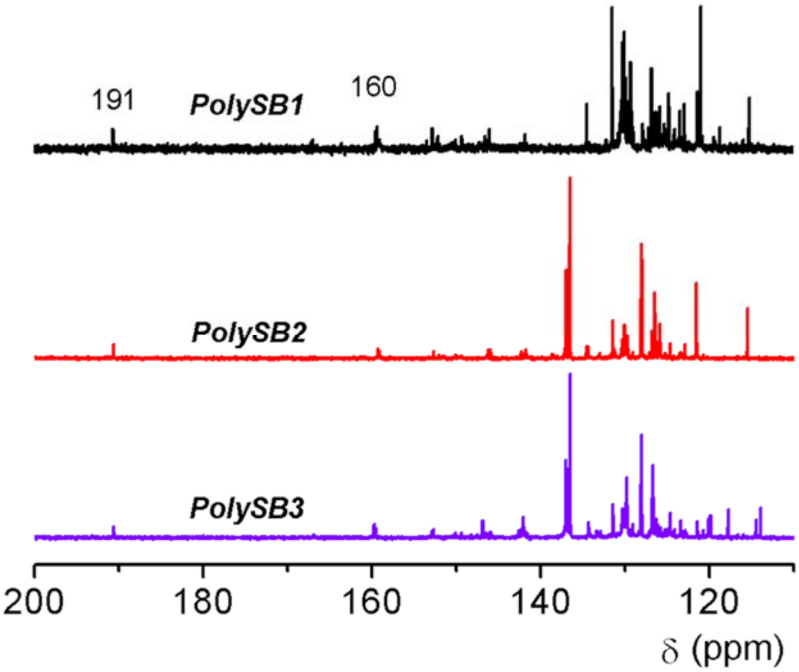


**Figure S5.** $^{13}C$ NMR spectra ($CDCl_3$, 101 MHz) of polySBs.

**Table S1.** Solubility of polySBs*.

| Polymer | $CHCl_3$ | $CH_2Cl_2$ | ACN | THF | Chlorobenzene | DMF | DMAc | DMSO |
|---|---|---|---|---|---|---|---|---|
| *polySB1* | + | + | ± | + | + | + | + | + |
| *polySB2* | + | + | ± | + | + | + | + | + |
| *polySB3* | + | + | ± | + | + | + | + | + |

Results were collected at c = 1 mg $mL^{-1}$ at RT. ±; partially soluble at RT but soluble in hot.

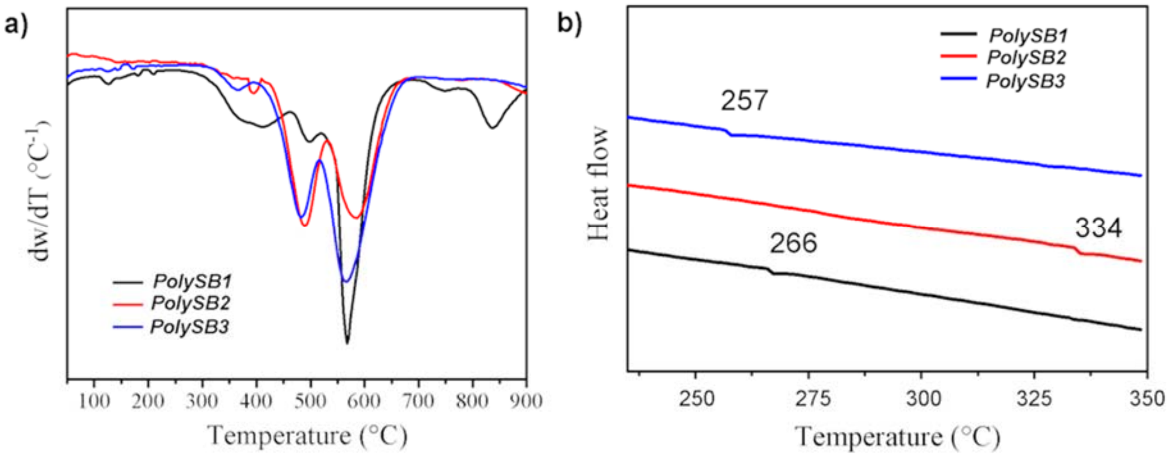


**Figure S6.** Thermal analysis of polySBs. **a**) DTGA and **b**) DSC.

**Table S2.** GPC results for the polySBs.

| Polymer | Mw (g mol$^{-1}$) | Mn (g mol$^{-1}$) | PDI [a] | DPn [b] |
|---|---|---|---|---|
| *polySB1* | 3882 | 1430 | 2.71 | 2.26 |
| *polySB2* | 4527 | 2083 | 2.17 | 2.65 |
| *polySB3* | 14849 | 2664 | 5.57 | 3.39 |

[a] Polydispersity index, estimated from Mw/Mn;

[b] Polymerization degree, estimated from Mn/molecular weight of the repeat unit.

**Photoluminescence measurements**

The emission behavior of polySBs were measured at room temperature. Samples were measured in diluted solutions and excited at their absorption maxima; 394 nm, 403 nm and 397 nm for polySB1, polySB2 and polySB3; respectively. The slits for the excitation and emission monochromators were equivalent to a bandwidth of 5.0 nm. PolySBs absorbances were close-fitted in the range of 0.11–0.14 (a.u.).

**Electrochemical methods**

Cyclic voltammetry (CV) measurements were performed on a three-electrode conventional three-compartment cell. As working electrode, a polycrystalline platinum disk (0.07 cm$^2$ geometric area) was used. A large area Pt coiled wire was used as counter electrode, which was separated from the electrolytic solution by a sintered glass. Before each measurement, the working electrode was polished with alumina slurry until a mirror finishing (particle size 0.3 mm), rinsed with water, and anh. dichloromethane (DCM). An Ag/AgCl electrode in *N,N,N*-trimethylmethanaminium chloride solution that matches the potential of an Ag/AgCl, KCl (1 M) electrode was used as reference electrode. Anhydrous dichloromethane was selected as solvent and tetrabutylammonium hexafluorophosphate (TBAHFP) as supporting electrolyte at a concentration of 0.1 M. TBAHFF was dried prior the use at 110 °C and kept into a desiccator. The concentration of polymer was adjusted to 1 mg mL$^{-1}$. Also, dry materials were employed for the manipulation of anh. dichloromethane maintained under a nitrogen atmosphere. Glassware was kept into an oven at 60 °C. Prior to each measurement, high purity nitrogen was flushed through the solution for 15 min, with the aim to remove any dissolved oxygen and an argon blanket was maintained over the solution during the measurements. All measurements were performed at room temperature.

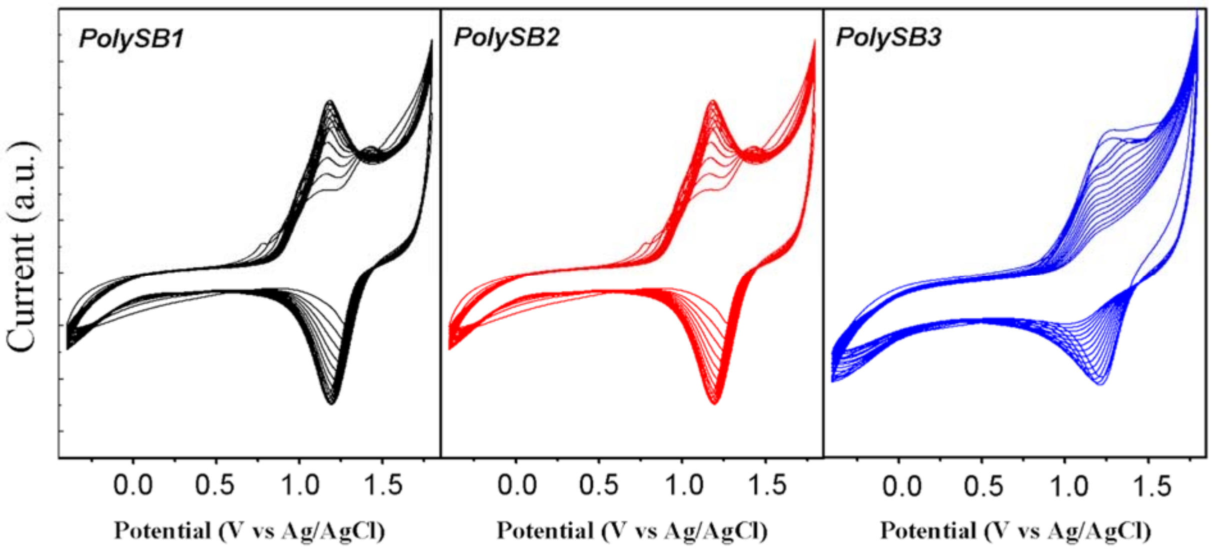


**Figure S7.** Cyclic voltammograms of polySBs at 15 cycles measurements in 0.1 M $Bu_4NPF_6$ in dichloromethane, at a scan rate of 100 mV $s^{-1}$.

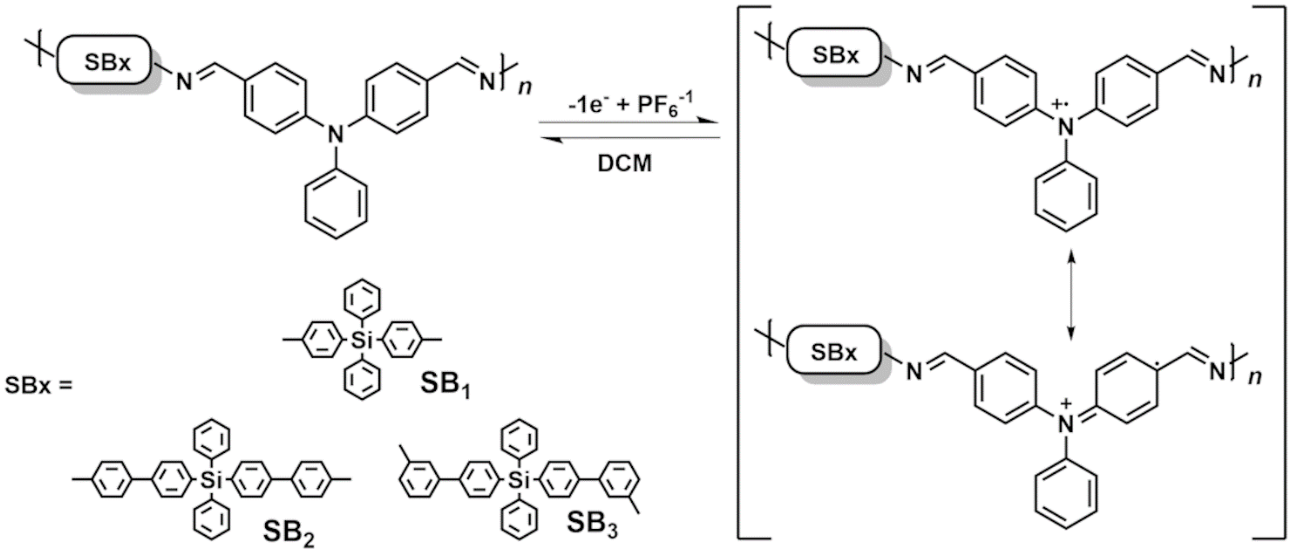


**Figure S8.** Redox process of the nitrogen atom from TPA-cores.
